# Adjuvant Radiation Therapy Alone for HPV Related Oropharyngeal Cancers with High Risk Features

**DOI:** 10.1371/journal.pone.0168061

**Published:** 2016-12-08

**Authors:** William Su, Jerry Liu, Brett A. Miles, Eric M. Genden, Krzysztof J. Misiukiewicz, Marshall Posner, Vishal Gupta, Richard L. Bakst

**Affiliations:** 1 Icahn School of Medicine at Mount Sinai, New York, New York, United States of America; 2 Department of Radiation Oncology, Mount Sinai Beth Israel, New York, New York, United States of America; 3 Department of Otolaryngology Head and Neck Surgery, Icahn School of Medicine at Mount Sinai, New York, New York, United States of America; 4 Department of Medical Oncology, Icahn School of Medicine at Mount Sinai, New York, New York, United States of America; 5 Department of Radiation Oncology, Icahn School of Medicine at Mount Sinai, New York, New York, United States of America; Fu Jen Catholic University, TAIWAN

## Abstract

**Background:**

Current standard of care for oropharyngeal cancers with positive surgical margins and/or extracapsular extension is adjuvant chemoradiotherapy. It is unknown whether HPV+ oropharyngeal cancer benefits from this treatment intensification.

**Objective:**

To investigate the outcomes of HPV+ patients treated with adjuvant radiotherapy alone when chemoradiotherapy was indicated based on high risk pathological features. They were compared with high risk HPV+ patients treated with adjuvant chemoradiotherapy.

**Methods:**

All high risk HPV+ oropharyngeal cancer patients (9) who received radiotherapy alone were identified. We also identified 17 patients who received chemoradiotherapy as a comparison group. Median follow up time was 37.3 months.

**Results:**

No local failures developed in adjuvant radiotherapy group. There was 1 distant recurrence in this cohort and 3 in CRT cohort. Regarding toxicity, 8 (47.1%) chemoradiotherapy patients had >10 lb. weight loss (p = 0.013), despite 75% of them having a percutaneous endoscopic gastrostomy tube placed. No individuals in radiotherapy group experienced a >10 lb. weight loss and none required a gastrostomy tube.

**Conclusions:**

This series provides preliminary evidence suggesting that the omission of concurrent chemotherapy to adjuvant radiotherapy may offer comparative local control rates with a lower toxicity profile in the setting of HPV+ patients with traditional high risk features.

## Introduction

Traditionally, definitive chemotherapy along with radiotherapy (RT) has been used to treat advanced or high risk oropharyngeal cancers (OPC). However, the emergence of transoral robotic surgery (TORS) has made minimally invasive surgical resection a possibility for select advanced cancers with small primary tumors (T1 and T2 disease). TORS is an alternative to standard definitive chemoradiotherapy (CRT) in appropriately selected patients, which results in lower surgical and post-operative RT related morbidity and toxicity as well as reducing the time to the start of post-operative adjuvant RT [1,2]. The growing recognition of TORS as an alternative to intensive CRT will likely result in an increase in the population of patients that will require adjuvant therapy. However, current adjuvant treatment guidelines are largely derived from clinical trials done in the pre-HPV era.

Extra-capsular extension (ECE) of positive lymph nodes and/or positive surgical margins are known to be high risk factors that traditionally warrant adjuvant CRT [3–5]. Two landmark randomized investigations completed by the Radiation Therapy Oncology Group and the European Organization for Research and Treatment of Cancer demonstrated the survival benefits of adding chemotherapy to adjuvant radiotherapy for OPC patients with these adverse pathologic findings [6,7]. Chemotherapy, which typically includes concurrent cisplatin, has been shown to be effective in OPC by acting as a radio-sensitizing agent [7,8]. The addition of chemotherapy to adjuvant radiation has improved survival in select patients but at a cost of increased acute toxicity [9].

HPV positivity is associated with more favorable prognoses consisting of better local control, longer overall survival, and better outcomes with standard treatment when compared to HPV negative disease [10–13]. Due to the improved prognosis of this patient population, there are a number of active studies investigating the possibility of treatment de-intensification to reduce toxicity [14]. A variety of strategies to de-intensify therapy are currently being evaluated and include: minimally invasive surgical protocols, reducing the radiation dose, reducing total radiation field, altered fractionation, alternative chemotherapy agents and dosages, and the omission of chemotherapy. Typical acute toxicity associated with adjuvant RT and CRT include mucositis, dysphagia, xerostomia, nausea and dysgeusia. Long term toxicities include feeding tube dependence, and pharyngeal or laryngeal dysfunction [15]. Due to dysphagia and nausea, the placement of a percutaneous endoscopic gastrostomy (PEG) tube is necessary for a significant number of patients who undergo this treatment.

Currently, there is no data regarding the use of radiation alone for treating ECE or positive surgical margins in HPV+ patients. A small study completed by Yokota et. al suggested the possibility of omitting chemotherapy in oropharyngeal cancers despite adverse pathological features [16]. However, they did not take HPV status into consideration. Another study suggested that adverse pathological features did not play a role in the development of loco-regional recurrence and distant metastases in HPV+ patients appropriately selected for TORS as their primary treatment [17]. This raises the question about whether chemotherapy can be omitted for these select patients. Herein, we sought to investigate loco-regional control rates and survival in a cohort of HPV+ patients who had pathological indications for adjuvant CRT, but received adjuvant RT alone.

## Materials and Methods

After receiving study approval from the Icahn School of Medicine at Mount Sinai Institutional Review Board, the radiation oncology database at our institution was queried for patients with early stage (T1-T3) OPC treated with TORS and neck dissection. A retrospective analysis of patient charts was completed. Their pathology reports were then reviewed to screen for HPV positive patients, leaving a cohort of 26 patients. At our institution, there are two steps to screening for HPV positivity. First, frozen sections are stained for p16, which is a surrogate marker for HPV. If tissue sample is p16+, polymerase chain reaction and in situ hybridization is completed to determine whether HPV is present and to identify the genotype of the virus. This ensures that patients are indeed HPV 16/18/31/33 positive. We then screened the pathology reports to identify HPV+ patients with high risk pathologic features including positive surgical margins and/or ECE.

Long term follow-up, disease status, survival, and toxicity complaints were determined by chart review. CTCAE 4.03 criteria were used to document the severity of certain toxicities, including xerostomia and mucositis. We considered those greater than or equal to grade 2 to be significant toxicity. Oncological surveillance was completed with physical exam, PET-CT and CT scans with contrast. Statistical analyses included Kaplan Meier survival, two-tailed t-tests, and chi-squared tests for equality of proportions and were completed by using R (version 2.15.2) along with the survival package.

## Results

### Patient Characteristics

26 total patients with HPV+ OPC and pathological indication for adjuvant chemoradiotherapy were identified. 1 (3.8%) patient was positive for HPV 31; the remaining patients were positive for HPV 16/18. Median follow up was 37.3 months and median age was 60 years old. These patients had positive ECE (13 patients), positive surgical resection margins (9 patients), or both (4 patients). There were 9 patients who were treated with adjuvant radiation alone, and 17 patients who were treated with adjuvant chemoradiotherapy. The 9 patients in our adjuvant RT alone group either refused to receive chemotherapy (6 patients) or were contraindicated for it due to co-morbidities (3 patients). Their median age was 66 years old and 5 (50%) had a positive smoking history. The remaining 17 patients all received adjuvant chemoradiotherapy. In this group, median age was 60 years old (p = 0.197) and 7 (41.2%) had a positive smoking history. Further patient characteristics, including TNM staging, are displayed in Table 1.

**Table 1:** Patient Characteristics.

	Chemo-Radiotherapy Population (17 Total)	Radiotherapy Only Population (9 Total)
**Age (p = 0.20)**	Median 60 years (range, 42–69)	Median 66 years (range 45–87)
**Sex (p = 0.21)**		
• Male • Female	• 16 (94.1%) • 1 (5.9%)	• 7 (77.8%) • 2 (22.2%)
**Positive Smoking History (p = 0.48)**	7 (41.2%)	5 (55.6%)
**Tumor Staging**		
• T1 • T2 • T3	• 6 (35.3%) • 11 (64.7%) • 0 (0%)	• 5 (55.6%) • 3 (33.3%) • 1 (11.1%)
**Nodal Staging**		
• N0 • N1 • N2	• 0 (0%) • 4 (23.5%) • 13 (76.5%)	• 1 (11.1%) • 2 (22.2%) • 6 (66.7%)
**Primary Tumor Location**		
• Base of Tongue • Tonsil	• 7 (41.2%) • 10 (58.8%)	• 1 (11.1%) • 8 (88.9%)
**Perineural Invasion**	3 (17.6%)	2 (20%)
**Lymphovascular Invasion**	5 (29.4%)	4 (40%)
**Neck Dissection**		
• Ipsilateral • Bilateral	• 15 (88.2%) • 2 (11.8%)	• 6 (66.7%) • 3 (33.3%)
**Radiation**		
• Ipsilateral Neck • Bilateral Neck	• 3 (17.6%) • 14 (82.4%)	• 6 (66.7%) • 3 (33.3%)
**Pathological Indication**		
• Extra-capsular Extension • Positive Surgical Margins • Both	• 9 (52.9%) • 5 (29.4%) • 3 (17.6%)	• 4 (44.4%) • 4 (44.4%) • 1 (11.1%)

### Treatment details

All patients in this study received intensity modulated radiation therapy (IMRT) to the neck. Further details regarding the radiation field given to the neck (ipsilateral vs bilateral) are shown in Table 1. Median radiation dose for adjuvant CRT group was 66 Gy (range 60–66 Gy) and 15 (88.2%) of 17 patients also received radiation to their primary tumor site. The majority were treated with concurrent weekly cisplatin (76.5%); detailed chemotherapy treatment regimens are given in Table 2. For adjuvant RT group, median radiation dose was 66 Gy (range 62–66 Gy) and 8 (88.9%) of 9 patients also received radiation to their primary tumor site.

**Table 2:** Treatment Regimens for Adjuvant Chemoradiotherapy Cohort.

Chemotherapy Received	Total (17 Patients)
Cisplatin	13 (76.5%)
Erbitux	1 (5.9%)
Taxotere and Erbitux	1 (5.9%)
5-Flurouracil, Hydroxyurea, and Erbitux	1 (5.9%)
Carboplatin and Taxol	1 (5.9%)

### Recurrences

There were 4 total recurrences in our patient population (Table 3). All recurrences were biopsy proven. Three were originally treated with adjuvant CRT and one was treated with adjuvant RT alone. In CRT group, all three recurrences were distant metastases. Within this cohort, one patient refused salvage therapy, one received salvage chemotherapy, and the remaining patient received salvage chemoradiotherapy. In the RT cohort, one of the patients experienced distant recurrence and received salvage chemoradiotherapy.

**Table 3:** Characteristics of Patients with Recurrent Disease.

Tx	Age (years) and Sex	Smoker	TNM	Primary Tumor	PNI	LVSI	Neck Dissection	RT Neck	High Risk Indication	Recurrence Location and Time
CRT	48M	-	T2N2M0	BOT	+	+	Ipslat	Bilat	ECE	Distant, 2.9 mo.
CRT	58M	-	T2N2M0	Tonsil	-	+	Ipslat	Bilat	ECE	Distant, 35.7 mo.
CRT	48M	-	T1N2M0	BOT	-	-	Ipslat	Bilat	ECE	Distant, 18.9 mo.
RT	66F	+	T2N2M0	Tonsil	+	+	Bilat	Ipsilat	ECE	Distant, 17.7 mo.

*Abbreviations****-*** Tx- Treatment Received, CRT- Adjuvant Chemoradiotherapy, RT- Adjuvant Radiotherapy, M- Male, F- Female, BOT- Base of tongue, PNI- perineural invasion, LVSI- lymphovascular space invasion, Ipsilat- ipsilateral, Bilat- Bilateral, ECE- extracapsular extension, mo.- months

### Survival

3-year disease free survival rate was 81.9% (95%CI, 65.3–100) for CRT group and 88.9% (95% CI, 70.6–100) for RT group. Overall, 2 of the 26 total patients have expired. One of these patients experienced recurrence and was from the cohort treated with adjuvant CRT. The other patient, also from the adjuvant CRT cohort, had complications arising from Hepatitis C liver cirrhosis. All patients who were treated with adjuvant RT alone are alive.

### Toxicity

Weight loss greater than 10 lbs. was common in the adjuvant CRT group. Eight patients (47.1%) experienced this, despite the majority of them (6 patients) having a PEG tube placed. Comparatively, no patients from the adjuvant RT experienced this magnitude of weight loss (p = 0.013). Furthermore, no patients in the adjuvant RT group required PEG tube placement. Meanwhile, 72.2% of the population receiving adjuvant CRT required or prophylactically opted for an acute PEG tube placement. Notably, one patient in CRT group was hospitalized twice due to nausea while receiving chemotherapy. Instances of acute and late toxicity from the study population are documented in Table 4.

**Table 4:** Toxicity.

	Adjuvant Chemoradiotherapy (17 total)	Adjuvant Radiotherapy (9 total)
**Acute Toxicity**		
• Weight Loss (>10 lbs.) p = 0.013 • Xerostomia (≥ Grade 2) p = 0.78 • Mucositis (≥ Grade 2) p = 0.59 • Dysgeusia p = 0.06 • Nausea p = 0.18	• 8 (47.1%) • 3 (17.6%) • 13 (76.5%) • 2 (11.8%) • 3 (17.6%)	• 0 (0%) • 2 (22.2%) • 6 (66.7%) • 4 (44.4%) • 0 (0%)
**Late Toxicity**		
• Xerostomia (≥ Grade 2) p = 0.70 • Dysphagia p = 0.78	• 7 (41.2%) • 3 (17.6%)	• 3 (33.3%) • 2 (22.2%)

## Discussion

This study suggests that adjuvant RT compared to adjuvant CRT may offer similar loco-regional control while potentially maintaining a lower acute toxicity profile in patients with HPV related OPC selected for transoral resection as the primary treatment modality. Some studies have suggested that HPV+ tumors are more sensitive to radiation [8]. It is possible that cisplatin, which is used as a radio-sensitizing agent, may not be necessary in select HPV+ tumors, because they are inherently responsive to radiation. Its selective omission could help address toxicity concerns and prevent the over-treatment of patients. The rate of distant metastases in HPV+ patients can remain high, despite control of local disease. This is consistent with previous studies [14,18]. In our cohort, 4 (15.4%) out of 26 patients experienced distant recurrence, with the lung being the most common site of recurrence. Concurrent cisplatin is often given at lower does with radiation as a radio-sensitizing agent, and as a single agent is not optimized to treat metastatic disease. Because of this, a difference in the rate of distant recurrence was not expected between the adjuvant CRT and RT groups.

Patients who received CRT tended to experience more severe nausea (p = 0.18), and weight loss (p = 0.013), despite the majority of patients having PEG tube placement. In the literature, it is well documented that the addition of chemotherapy to radiation treatment regimens leads to increased acute treatment related toxicity along with late toxicity [15, 19, 20]. Some toxicity could be prevented by reducing the amount of chemotherapy that these patients usually receive or omitting it completely. Furthermore, choice in feeding tube placed may also help address long term dysphagia that patients experience [21].

The number of patients who receive adjuvant RT alone when pathologically indicated for CRT are low, because these patients were not receiving standard of care based on their disease characteristics at the time of treatment. Our study was not adequately powered to compare survival differences between our two treatment groups. CRT group had a higher proportion of patients with base of tongue cancers and receive bilateral neck radiation. These treatment differences limit the conclusions that can be drawn from this comparison. However, it should be noted that all sub-groups within CRT group were experiencing similar toxicity. While toxicity data was readily available for weekly visits and long term follow up, the retrospective nature of this study has inherent limitations to reliably comparing the toxicity amongst treatment groups. Furthermore, patients who did not receive chemotherapy because of co-morbidities are already a higher risk population and might be more susceptible to recurrence or other forms of treatment related toxicity. Co-morbidities such as older age, smoking and alcohol history adversely affect prognosis [22].

There are a variety of ongoing trials investigating treatment de-intensification in this population, such as ECOG 3311. At our institution, we are currently enrolling HPV+ patients with ECE and/or positive surgical margins in a clinical trial for treatment de-intensification consisting of 56 Gy of radiation along with concurrent cisplatin [23]. Our current results raise the question about whether adjuvant RT alone is sufficient in this high-risk surgical population. This study provides preliminary data to design future trials to determine optimal adjuvant regimen for HPV positive disease.

## Conclusions

For HPV+ OPC patients with high-risk features, adjuvant RT alone potentially offers comparative local control rates with a more favorable acute toxicity profile. This data suggests that one method of treatment de-intensification may be the utilization of adjuvant RT alone for patients with ECE or positive surgical margins. Further studies are warranted to determine the long-term efficacy, safety, toxicity, and optimal radiation dosing in this setting.
